# Harvi Cardiovascular Modeling Accurately Predicts Hemodynamic Improvements Produced by a New Direct Cardiac Compression Device

**DOI:** 10.1097/MAT.0000000000002346

**Published:** 2024-11-22

**Authors:** Erica C. Perez, Christina M. Bolch, Reagan M. Tompkins, Daniel Burkhoff, George V. Letsou, John C. Criscione

**Affiliations:** From the * Department of Biomedical Engineering, Texas A&M University, College Station, Texas; † Department of Engineering, CorInnova, Inc., Houston, Texas; ‡ Division of Heart Failure, Hemoydnamics and MCS Research, Cardiovascular Research Foundation, New York, New York; § Department of Surgery, University of Houston Medical School, Houston, Texas; ¶ TransMedics, Inc., Andover, Massachusetts; ‖ School of Engineering Medicine, Texas A&M College of Medicine, Bryan, Texas.

**Keywords:** mechanical circulatory support, direct cardiac compression, heart failure, Harvi, simulation

## Abstract

Despite advancements in mechanical circulatory support (MCS) technology, persistent critical complications related to blood contact remain unresolved. To provide a safer alternative therapy, CorInnova is developing a non-blood contacting direct cardiac compression (DCC) device for MCS. To support product development toward clinical trials, a simulation platform has been developed to predict clinical outcomes under patient-specific conditions, guiding patient selection for clinical trials. The Harvi simulation was validated using preclinical *in vivo* data from experimental studies with the CorInnova device, with n = 28 hemodynamic samples simulated from animal data (n = 4 ovine). After confirming validation, further simulation was performed to predict additional hemodynamic outcomes not captured in animal studies. The simulated effects of CorInnova device therapy were not significantly different from animal data for cardiac output, systemic arterial blood pressure, mean pulmonary artery pressure, central venous pressure, or left ventricular pressure (*p* > 0.050). Harvi accurately predicts the effects of the CorInnova device in heart failure conditions and can be used in preparation for future clinical trials.

## Graphical abstract

**Figure:**
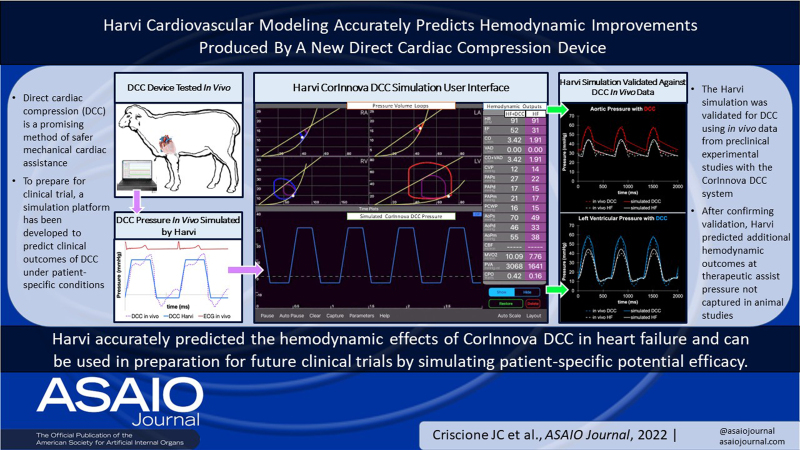

Heart failure (HF) is a chronic debilitating condition with a globally increasing burden. In the USA, prevalence is expected to increase by 46% (2012–2030), affecting greater than eight million adults.^1^ When HF medical management is insufficient, mechanical circulatory support (MCS) devices can be applied temporarily as bridge therapy or chronically implanted. Mechanical circulatory support therapy has been used for decades with iterative improvements, with some devices being recalled by regulatory bodies due to patient safety concerns.^2^ Many currently available devices are highly invasive, involving surgical placement within the vascular system and anticoagulation with its attendant risks. These risks include stroke, hemorrhage, vascular injury, neurologic injury, bleeding, and renal dysfunction.^3–7^ Extracardiac implanted devices have the potential to minimize risks associated with MCS adverse events. An extracardiac device that applies direct cardiac compression (DCC) (CorInnova, Inc, Houston, TX) is completing animal trials with human clinical trials planned. The CorInnova device applies cyclic positive and negative pressure directly to the heart’s ventricular surface in synchrony with the native heartbeat, augmenting cardiac systolic and diastolic performance.^8–10^ An illustration of the CorInnova implantable is shown in Figure 1.

**Figure 1.**
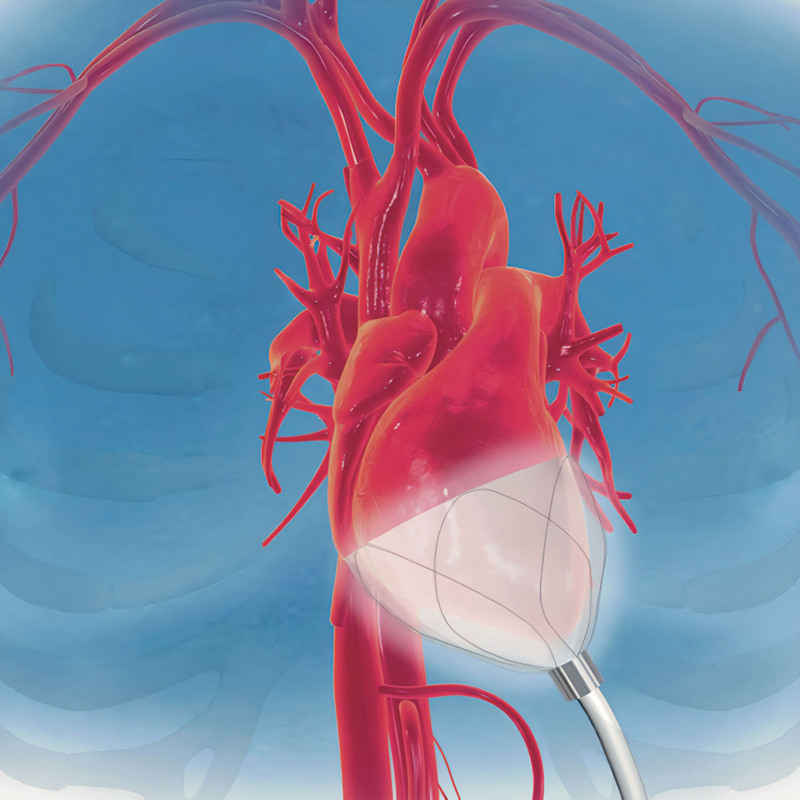
Concept illustration of the CorInnova DCC heart assist device, currently in development. DCC, direct cardiac compression.

Cardiovascular simulation is helpful for MCS device research and development.^11–14^ Simulations are useful during preclinical testing to predict hemodynamic effects on targeted clinical patient populations which may aid in planning clinical trials. Simulations may also be used to predict the effects of MCS on individual patients with specific HF phenotypes.^15^ The Harvi simulation platform (Harvi Dynamics, Inc. Remsenburg, NY) is a validated system that provides such hemodynamic models, easily accessible from tablets or a web browser.^16,17^ The Harvi simulation is widely used to describe and, in some cases, predict the effects of device and drug therapeutic interventions on hemodynamics based on patient data.^11–13,15,18–20^

We examined the accuracy of the Harvi simulation platform in predicting the experimental results of the CorInnova DCC device in animals with HF. Furthermore, we used the platform to predict device performance with changes in device operation settings. We sought to validate that Harvi accurately predicts *in vivo* effects of the CorInnova DCC cardiac assist device so that it may be used to assess a newer, potentially safer MCS therapy.

## Materials and Methods

### Experimental Assessment of the CorInnova Cardiac Assist Device in an Ovine Heart Failure Model

All applicable international, national, or institutional guidelines for the care and use of animals were followed. Experiments were conducted under an animal use protocol that was approved by the Texas Heart Institute (AUP# 2014-11) or the Texas A&M University Institutional Animal Care and Use Committee (AUP# TIPS-01014). Studies were performed in four sheep in which biventricular (BiV) HF was induced with high-dose esmolol.^8^ The hemodynamic profile of the animals was characterized by continuous measurement of heart rate (HR), aortic blood pressure (AoP), pulmonary artery pressure (PAP), ascending aortic flow (AoF) for measuring cardiac output (CO), central venous pressure (CVP), and left ventricular pressure (LVP). Hemodynamics were assessed before esmolol, during esmolol-induced HF and, finally, during esmolol HF with cardiac support provided by the CorInnova DCC device. Hemodynamic measurements were intentionally paired with successive DCC measurements to minimize the effects of esmolol’s short half-life. This experimental group of four animals generated the hemodynamic data used to validate Harvi’s accuracy, both for HF alone and for HF during assist with the CorInnova device. Animal data are summarized in Table 1.

**Table 1.** Average Hemodynamic Simulation Key Inputs for “Healthy” Baseline and HF Conditions, Obtained From Animal Data

Average Simulator Inputs From *In Vivo* Data			
Hemodynamic Parameter	Units	Baseline (n = 4)	HF (n = 12)
Heart rate	min^−1^	109 ± 18	91 ± 3
LV ejection fraction	%	50 ± 0	29 ± 3
Cardiac output	L/min	4.09 ± 1.00	1.91 ± 0.22
Central venous pressure	mm Hg	10 ± 3	14 ± 2
Pulmonary capillary wedge pressure	mm Hg	13 ± 3	15 ± 2
Pulmonary artery pressure	mm Hg		
Systolic		24 ± 5	22 ± 3
Diastolic		13 ± 3	14 ± 3
Mean		17 ± 3	16 ± 2
Aortic pressure	mm Hg		
Systolic		82 ± 7	49 ± 3
Diastolic		60 ± 12	33 ± 5
Mean		67 ± 10	38 ± 4

HF, heart failure; LV, left ventricular.

### Harvi Cardiovascular Simulation

The Harvi cardiovascular simulation platform was customized to include a module simulating the hemodynamic effects of DCC according to data obtained from prior isolated canine experiments.^17,21–23^ In brief, contractile properties of each cardiac chamber were modeled as a time-varying elastance, which has been validated for both the ventricles and for the atria.^11^ The systemic and pulmonary vascular beds were modeled by a series of resistance and capacitance elements. Parameters of the model were adjusted to fit the hemodynamic profile of the animals at the baseline “healthy” and HF states, before DCC activation. Variable model parameters included standard hemodynamic data (Table 1), as well as stressed blood volume, systemic and pulmonary afterload (including arterial resistance [Ra], characteristic impedance [Rc], and arterial compliance [Ca]), systolic properties (end-systolic elastance [E_es_]) of each of the four heart chambers, ventricular Tmax (time to end-systole), and the time constant of ventricular relaxation (tau [*τ*]). The equations forming the foundation of Harvi have been provided previously.^24–27^ With Harvi, a user is able to quickly simulate a subject’s hemodynamic profile and then predict the effects of therapeutic interventions.

### Harvi Simulation of CorInnova Direct Cardiac Compression

**Figure 2.**
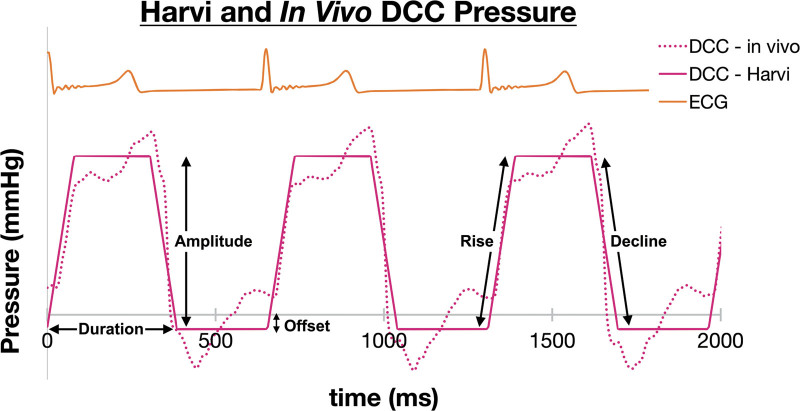
Comparison of *in vivo* (dotted magenta) to Harvi (solid magenta) of the CorInnova direct cardiac compression assist pressure waveform, illustrating the parameters used to simulate CorInnova DCC; the CorInnova system operates in synchrony with the ECG (orange). DCC, direct cardiac compression; ECG, electrocardiogram.

Harvi simulates DCC by adding the compression pressure to the left ventricular (LV) and right ventricular (RV) time-varying pressures otherwise determined from the model.^23^ The CorInnova device’s active assist chambers cyclically inflate and deflate in synchrony with the heart, and are controlled by the drive system. CorInnova DCC device compression pressure was approximated *in silico* as a trapezoidal waveform with a user-defined inflation rate (“Rise”), a constant peak pressure applied during systole, deflation rate (“Decline”), and a constant negative deflate pressure applied during diastole (“Offset”). These model inputs controlling the simulated DCC assist pressure are illustrated in Figure 2, with comparison to an example *in vivo* DCC assist pressure waveform. The full list of CorInnova DCC simulation settings is reported in Table S1 Supplemental Digital Content, http://links.lww.com/ASAIO/B357.

### Harvi Validation Study

For baseline “healthy” (pre-HF) and HF simulations, Harvi parameters were adjusted until hemodynamics generated by the model matched the values measured in the ovine study; to ensure optimal *in silico* reproduction of ovine conditions, the acceptance criteria for simulation outputs were: 1) equivalent circulation metrics, and 2) *in silico* pressures within ±2 mm Hg of *in vivo* pressure. The effects of CorInnova DCC at each of the specified device settings (matching animal studies) were then predicted using Harvi. Effects predicted by the model were then compared to those measured in the experimental animals for validation of Harvi.

For each of the four animals, seven data sets were simulated: one baseline “healthy” data set (*ie*, pre-HF conditions), three HF data sets without support from DCC (HF), and three HF data sets with CorInnova DCC (HF + DCC). In total, 28 states were simulated: 4 baselines, 12 in HF, and 12 HF + DCC. Pulmonary artery catheter (PAC) data were only available from two animals. The data used for model inputs for baseline and HF samples are summarized in Table 1 (mean ± standard deviation), with additional model parameters summarized in Table S2, Supplemental Digital Content, http://links.lww.com/ASAIO/B357. The baseline data sets were used only to identify normal simulation values for each animal.

To quantitatively validate the simulated effect of CorInnova DCC assist during HF compared to *in vivo* effects, the absolute change in each hemodynamic parameter between HF and HF + DCC was recorded from *in vivo* and *in silico* (∆hemodynamics). This permitted indexed averaging of the device effect on hemodynamics across differing device settings and various animal cardiovascular states over the study population (n = 12). Two-tailed Student’s t-tests (α = 0.050) were performed to test for significant differences between *in vivo* and *in silico* hemodynamic parameters during baseline and HF conditions, and to test for significant differences between observed and simulated effects of DCC.

### Simulation of CorInnova Direct Cardiac Compression Pressure-Volume Loops

After validation of the CorInnova DCC module in Harvi with *in vivo* data, simulated RV and LV pressure-volume (PV) loops with end-systolic PV relationships (ESPVR) and end-diastolic PV relationships (EDPVR) were generated. These PV loops and resulting EDPVRs and ESPVRs were used to assess the potential effects of CorInnova DCC beyond what has been studied *in vivo*. The chambers of the CorInnova device can be inflated to provide varying amounts of support. We used two different DCC simulation pressures: 19 and 30 mm Hg, identified by “HF + DCC19” and “HF + DCC30.” A 19 mm Hg DCC pressure provides effective assist (representative of the average of animal studies), and 30 mm Hg DCC pressure provides additional assist without exceeding RV systolic pressure that might compromise RV performance. Thus, the additional HF + DCC30 simulation with an input of 34 mm Hg for the amplitude and 4 mm Hg as the diastolic offset was performed. All simulation settings for both “HF + DCC19” and “HF + DCC30” are summarized in Table S1, Supplemental Digital Content, http://links.lww.com/ASAIO/B357.

## Results

### Validation of Harvi Cardiovascular Simulation

To confirm that Harvi with DCC modification represents the *in vivo* results accurately, we first confirmed Harvi’s ability to reproduce the baseline hemodynamics and the hemodynamics after induction of HF. As summarized in Table 2, the average *in silico* hemodynamic parameters obtained from modeling each animal’s baseline and HF data were not significantly different (*p* > 0.050) than *in vivo* (Table 1). Likewise, there were no significant differences between the simulated HF and the animal HF data (*p* > 0.050), aside from an acceptable difference of 2 mm Hg in pulmonary artery diastolic pressure (PADP) (*p* = 0.040).

**Table 2.** Summary of the Average Simulation Outputs for Baseline, HF, and HF + DCC

Average Simulation Results: Baseline, HF, HF + DCC				
Simulation Output Parameter	Units	Baseline (n = 4)	HF (n = 12)	HF + DCC (n = 12)
Aortic pressure	mm Hg			
Systolic		82 ± 7	50 ± 4	63 ± 7
Diastolic		60 ± 12	33 ± 5	41 ± 8
Mean		67 ± 10	38 ± 4	49 ± 7
Pulmonary artery pressure	mm Hg			
Systolic		24 ± 5	22 ± 3	25 ± 4
Diastolic		13 ± 4	16 ± 2*(*p* = 0.040)	17 ± 2
Mean		16 ± 5	18 ± 2	20 ± 2
Pulmonary capillary wedge pressure	mm Hg	13 ± 3	15 ± 2	16 ± 3
Central venous pressure	mm Hg	10 ± 3	14 ± 2	13 ± 3
LV EDP	mm Hg	12 ± 4	15 ± 2	15 ± 3
LV SV	mL	38 ± 11	21 ± 3	31 ± 3
LV total cardiac output	L/min	4.08 ± 1.17	1.92 ± 0.24	2.80 ± 0.21

* *p* < 0.050 indicates potentially statistically significant difference from *in vivo* data, however, all baseline and HF simulations met acceptance criteria.

EDP, end-diastolic pressure; HF + DCC, heart failure with direct cardiac compression support; LV, left ventricular; SV, stroke volume.

**Figure 3.**
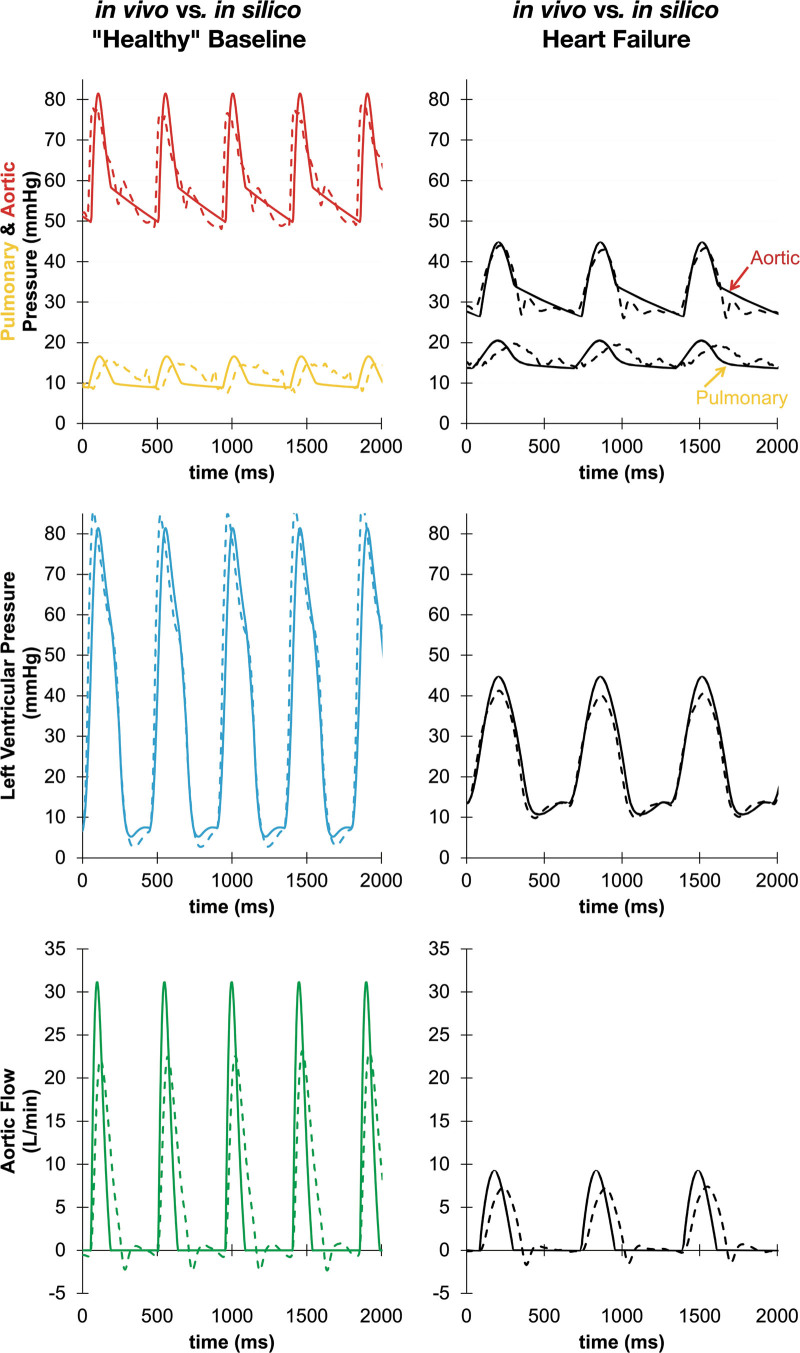
Hemodynamic waveforms from a representative animal (*in vivo*) compared to the resulting Harvi simulation of the animal’s hemodynamic data (*in silico*); colored lines show the baseline conditions where the black lines show the heart failure conditions; the dotted lines are the *in vivo* animal waveforms, and the solid lines are the resulting Harvi-simulated waveforms. Note the likeness between *in vivo* and *in silico.*

In addition to the quantitative metrics, the Harvi-simulated hemodynamic waveforms were comparable to waveforms observed in the animals. An example comparison of the Harvi and observed waveforms is shown in Figure 3.

### Validation of CorInnova Direct Cardiac Compression Heart Assist Simulation

**Figure 4.**
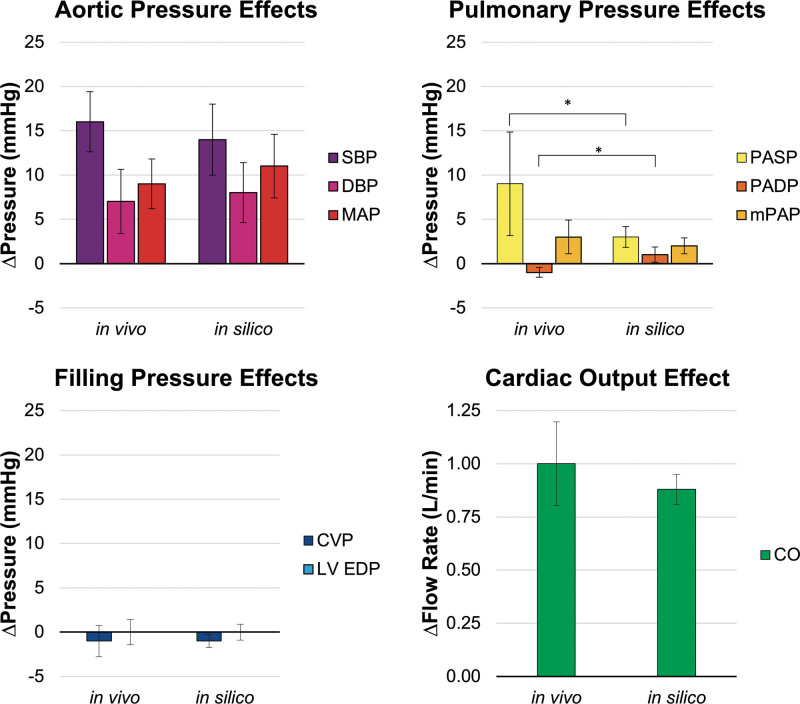
Summary of the absolute effects on key hemodynamic parameters (∆hemodynamics) resulting from heart assist with the CorInnova direct cardiac compression device (HF + DCC) used to validate the Harvi model DCC predictions; the actual effect (*in vivo*) is compared graphically here to the simulated effects (*in silico*); results showed that the simulated hemodynamic effects from CorInnova DCC predicted by Harvi did not differ significantly from the *in vivo* effects, aside from systolic (PASP, *p* = 0.028) and diastolic (PADP, *p* = 0.001) pulmonary pressures (*p < 0.050); yet, the mPAP did not differ significantly thus the model was considered validated for this scope. CO, cardiac output; CVP, central venous pressure; DBP, diastolic blood pressure; DCC, direct cardiac compression; HF, heart failure; LV EDP, left ventricular end-diastolic pressure; MAP, mean arterial pressure; SBP, systolic blood pressure.

To validate the CorInnova DCC module addition to Harvi, each animal-specific, *in silico* HF + DCC simulation was compared to observed hemodynamic waveforms generated in the *in vivo* HF + DCC studies. Figure 4 summarizes the *in vivo* and *in silico* effects of DCC on hemodynamics. There were no significant differences between *in silico* and *in vivo* results for aortic pressures (systolic [SBP], diastolic [DBP], mean [MAP]), CVP, LV end-diastolic pressure (LVEDP), nor CO (all *p* > 0.050). The Harvi-simulated *in silico* effect of assist on the pulmonary artery systolic (PASP, *p* = 0.028) and diastolic (PADP, *p* = 0.001) blood pressure trended toward a significant difference from *in vivo* (*p* < 0.050); the measured *in vivo* PAP increased more during systole and mildly decreased during diastole with CorInnova DCC assist, whereas the Harvi-predicted effect on PASP/PADP was mild. However, there was no significant difference in mean PAP (mPAP, *p* = 0.426) between *in silico* and *in vivo* (*p* > 0.050). Harvi accurately predicted that for a given set of n = 12 CorInnova assist settings applied during esmolol HF, on average the MAP increased by +11 mm Hg, mPAP increased by +2 mm Hg, CVP decreased −1 mm Hg, LVEDP did not change, and CO increased +0.88 L/min.

**Figure 5.**
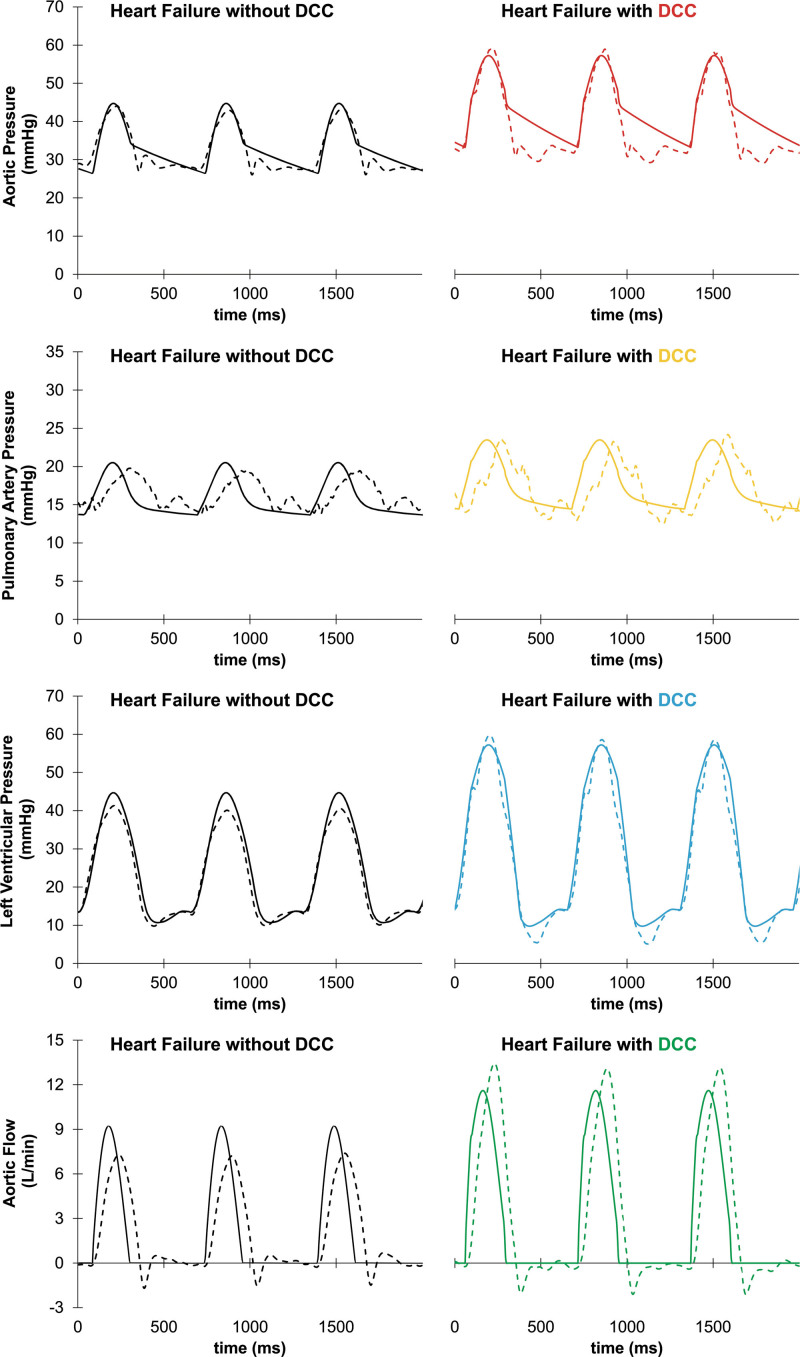
Representative hemodynamic waveforms demonstrating comparable waveform morphology between *in vivo* and *in silico* during HF and CorInnova direct cardiac compression during HF (HF + DCC); colored lines show the results of HF + DCC, with black showing HF before activating DCC support; dashed lines show actual *in vivo* waveforms, and solid lines show Harvi-simulated waveforms.

The Harvi HF + DCC waveforms also closely mimic the *in vivo* HF + DCC waveforms. The colored lines in Figure 5 represent waveforms resulting from Harvi (solid) versus *in vivo* (dashed), compared to the unassisted HF waveforms (black).

### Simulation of Increased CorInnova Device Assist Pressure and Pressure-Volume Loop Assessment

**Figure 6.**
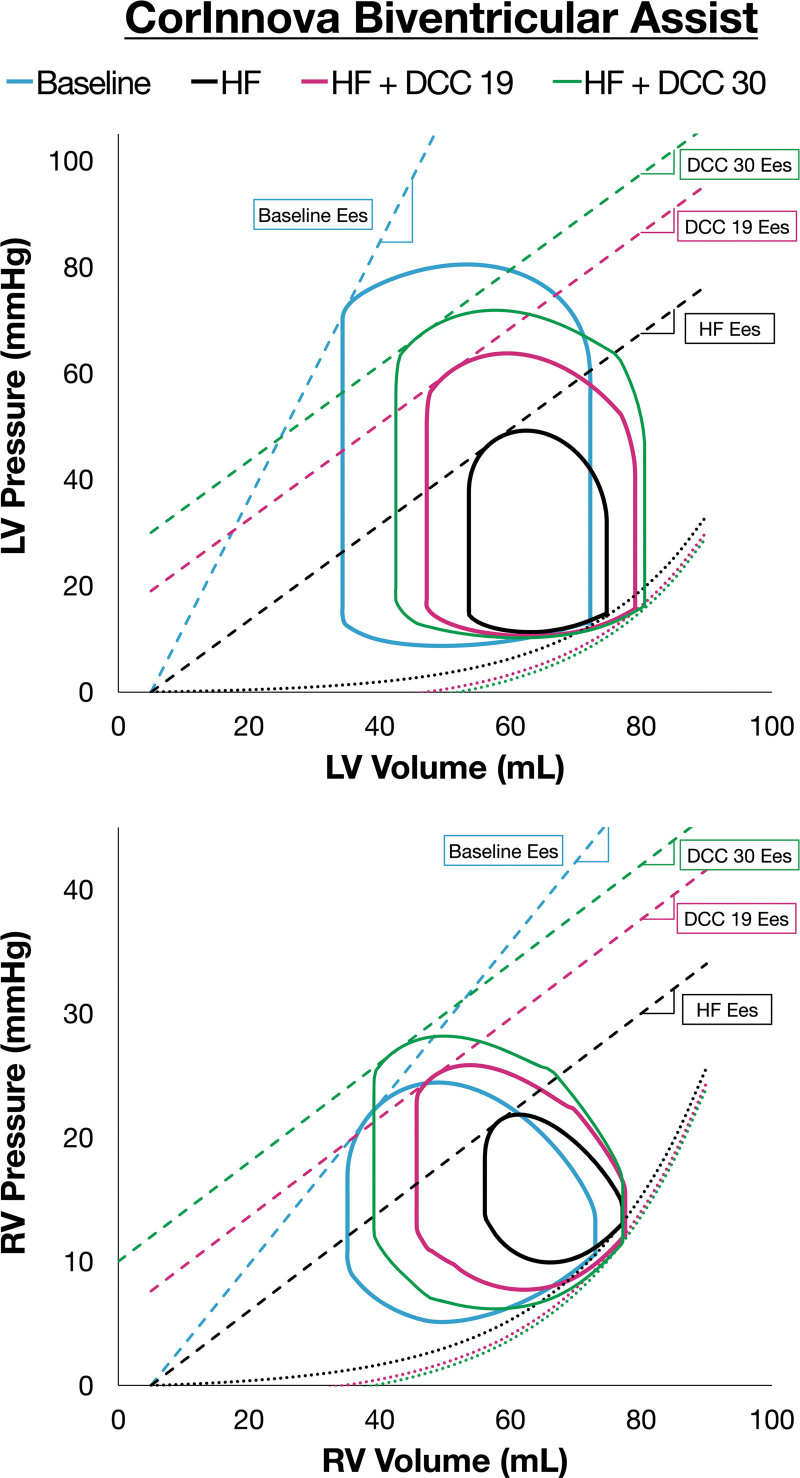
LV and RV PV loops for baseline (cyan), HF (black), and CorInnova direct cardiac compression during HF (HF + DCC); HF + DCC PV loops were simulated at 19 mm Hg (HF + DCC19, magenta) and at 30 mm Hg (HF + DCC30, green); each ESPVR is a dashed line with Ees (slope) labeled, and each EDPVR is a dotted curve. DCC, direct cardiac compression; EDPVR, end-diastolic pressure-volume relationships; Ees, end-systolic elastance; ESPVR, end-systolic pressure-volume relationships; HF, heart failure; PV, pressure-volume.

Figure 6 illustrates RV and LV PV loops simulated for baseline (cyan) and HF (black) generated using data averaged from all four animals. Comparing HF to baseline, Harvi-simulated RV and LV PV loops significantly reduced in height and narrowed with the reduction in SV, predominately on the left side as the end-systolic volume (ESV) decreased significantly, with less change in the end-diastolic volume (EDV). End-diastolic pressure-volume relationships were constant between baseline and HF, with loops shifted to the right along the EDPVR with mild increases in LVEDP (+2 mm Hg) and RVEDP (+3 mm Hg) relative to baseline.

Right ventricular and LV PV loops are also plotted for two different assist level settings of the CorInnova DCC device: 19 (magenta) and 30 (green) mm Hg. When HF + DCC was simulated, PV area (PVA) increased with increasing SV and SBP; PV loops shifted toward the pre-esmolol “healthy” baseline loops. End-systolic pressure-volume relationship slope (E_es_) did not change but the ESPVR shifted upward in response to DCC, with the magnitude of the shift equal to the peak of the systolic assist pressure (19 or 30 mm Hg).

## Discussion

Preclinical testing of cardiac assist devices in animal models is an important aspect of product development, but animal models may not precisely predict device performance in humans. Computer simulations of cardiac performance, such as Harvi, are gaining increasing importance in product development. Computer simulation platforms also provide physicians with real-time hemodynamic predictions of various HF treatment strategies. These predictions can aid appropriate patient selection for clinical use of different devices and aid in clinical trial design of new medical devices.

Harvi models the cardiovascular system as a collection of resistors, capacitors, and diodes and each of the heart chambers as a time-varying elastance.^11,28–30^ The model has been applied for clinical simulation of experimental and clinically available MCS devices, such as peripheral bypass pumps, transvalvular pumps, and implantable ventricular assist devices (VADs). The model has been shown to predict hemodynamic effects of therapies in different patient scenarios.^11–13,15,18^ The primary advantage of this approach to modeling is the ability to predict hemodynamic effects in real time without extensive computational power. Harvi is easily accessible on a tablet or browser with user inputs from readily available bedside information (*ie*, vital signs, hemodynamics, and echocardiogram measurements).

In the current study, Harvi accurately predicted CorInnova DCC device effects observed in animal experiments. Systolic blood pressure, DBP, MAP, mPAP, CVP, LVEDP, SV, and CO were all accurately predicted. In addition to the hemodynamic parameters, it is remarkable that the *in vivo* and *in silico* hemodynamic waveforms were so similar (Figures 3 and 5). Any minor differences may be due to measurement artifact *in vivo* that is not replicated by a simulation, or potentially imperfect replication of vascular properties we cannot directly measure (*eg*, wall tissue mechanics, blood volume). There is a statistically significant difference between the simulated and actual effects of CorInnova DCC on PASP and PADP according to the t-test results (*p* < 0.050). However, the standard deviation from the mean in these groups was relatively large, and the small differences observed in the PAP parameters are possibly due to the limited *in vivo* PAC measurements. Moreover, the difference between mPAP *in vivo* versus mPAP *in silico* was not statistically significant.

Understanding the effect of device therapy on hemodynamics and four-chamber PV relations is essential to an informed assessment of HF patient-specific device effect—leading to optimal patient outcomes. Thus, a modern tool with accurate PV loop predictions in addition to comprehensive hemodynamics is highly valuable. In the current study, validation of the simulated hemodynamic data enabled further assessment of CorInnova DCC beyond what has been tested *in vivo*. Comparing HF to baseline, the changes in PV loops were consistent with the anticipated negative-inotropic effect from esmolol, which primarily affected systolic contraction (ventricular elastance, E_es_). Furthermore, maintenance of the same EDPVR curve is expected in this HF model because ventricular compliance does not acutely change with esmolol infusion. When HF + DCC was simulated, PV loops shifted toward the pre-esmolol baseline loops. The parallel ESPVR shift upward in response to DCC equal to the peak of the systolic assist pressure waveform is consistent with previous experimental work demonstrating that E_es_ is acutely unchanged by DCC.^21–23,31^

The BiV increase in loop size with CorInnova DCC is primarily due to a reduction of ESV and increased systolic pressures, demonstrating BiV recruitment of additional stroke work (SW) with device support; unique to DCC, this additional SW is attained by the device, with no additional work done by the heart. *In silico* LVEDP was mostly unchanged by DCC in this HF model, however RVEDP declined by −2 mm Hg with 30 mm Hg DCC. Additionally, each HF + DCC RV loop demonstrated some early diastolic filling assistance, approaching the baseline RV PV loop. Importantly, the increase of RV peak pressure on the PV loop is less than that for the LV loop and remains within physiologic range for RV systolic pressure, despite uniform BiV epicardial assist.

For both ventricles, CorInnova DCC assist with 19 mm Hg results in 84.2% recovery of baseline SV, and 30 mm Hg assist results in 100% recovery of the baseline SV. This prediction of full recovery of baseline SV supports the CorInnova system’s potential to provide sufficient pulsatile circulatory support in patients with circulatory collapse. In addition to maintaining physiologic pressure and blood flow, valve function is maintained and recovery to the baseline PV loop morphology is facilitated with the CorInnova device, with the DCC loop approaching that of the “healthy” baseline PV loop. This ventricular PV loop shift predicted by Harvi is a unique result of DCC cardiac assist. For comparison, Impella (Abiomed; Danvers, MA) devices result in a more triangular shape of the ventricular PV loop and, in the setting of severe ventricular contractile dysfunction, a complete separation between LVP and AoP as normal aortic valve opening is absent with Impella full ventricular unloading.^13,32^ Direct cardiac compression cardiac assist preserves PV loop morphology and aortic valve opening. Maintenance of natural ventricular motion is an objective of the CorInnova device and may facilitate intrinsic mechanobiological processes linked to improved ventricular wall motion and geometry.^33^

In our study, Harvi PV loops demonstrated mildly increased EDV during CorInnova DCC application. However, *in silico* studies of other DCC prototypes conducted using alternative cardiovascular simulation models predicted a decline in EDV with DCC ventricular assist—similar to results from experiments in isolated canine hearts with a previous generation of DCC.^34,35^ Reduced EDV may be clinically beneficial in severely dilated hearts with high wall stress, as simulated by Aranda-Michel *et al*^34^; however, the animal data simulated in our study was a pharmacological model of acute hypotensive HF with depressed contractility, low afterload, and relatively low preload in non-dilated hearts that, aside from esmolol, were otherwise healthy. Similar animal conditions were simulated by the “*in silico* twin” of the AdjuCor BEAT DCC.^35^ Even in their “low-afterload HF” conditions, DCC with BEAT resulted in mildly reduced EDV. Our differing results with Harvi-CorInnova may result from differing model assumptions, variability in experimental HF, and differing DCC systems.

Familiarity with Harvi is growing within the clinical, experimental, and academic communities. Harvi is routinely used to explore and demonstrate complex hemodynamics resulting from medical and MCS therapies. Our results confirm that Harvi can replicate hemodynamic metrics in normal and experimental HF states as well as during DCC MCS. These results demonstrate that Harvi can be used to simulate specific patient profiles and predict the acute hemodynamic effects of the CorInnova DCC device, which may be helpful for patient selection in clinical studies and to identify responders in clinical practice. If scalable to human hearts, the results show that the degree of support can be effective in patients with severe BiV failure. Our results in this acute HF model highlight improved systemic blood pressure, LVP, SV, and inotropic effect d(LVP)/d(t)—all beneficial to an acutely decompensated HF patient for whom inotropic and vasopressor support has failed to sufficiently support end-organ function and a path toward recovery or other positive outcome. It remains to be seen how hemodynamic effects of DCC may vary under high vascular resistance or normotensive or hypertensive conditions; this can be examined experimentally *in vivo* and *in silico* with the now-validated Harvi. We can speculate that we may see a reduction in high preload and improved SV with reduced EDV in these patients. Clinically, the CorInnova device may be particularly useful in patients with contraindications to other devices, such as bleeding preventing anticoagulation, poor vascular access, or aortic valve insufficiency. The need for continued MCS device innovation, particularly the call for non-blood contacting support, has been recently reviewed.^36^ It is essential that we continue to develop new technology to address the unresolved complications associated with current MCS devices.

### Study Limitations

This simulation study was limited by the number of available animal data samples, particularly the PAC data. Future studies with high-quality imaging will be needed to confirm the volume simulation. This simulation of DCC heart assist was an effective approximation but does not perfectly reflect the actual pressure waveform; the actual deflate duration is brief enough to evacuate the compression bladders and does not hold suction for the entire duration of diastole. Nevertheless, the simulated CorInnova DCC outcomes were validated by the real *in vivo* data.

## Conclusions

Simulation of the cardiovascular system with Harvi offers a way to predict the complex hemodynamic effects of MCS in patients with HF. This study confirms Harvi’s accuracy of DCC with the CorInnova device using preclinical animal study parameters and results. The simulations confirm the observed hemodynamic benefits of the CorInnova DCC device in an animal HF model. Harvi quickly simulates a subject’s hemodynamic profile and can then predict real-time effects of therapeutic interventions such as DCC. We expect Harvi simulations to play an important role in planning the CorInnova clinical trial and in identifying appropriate patients for DCC.

## Supplementary Material

## References

[1] Martin SS Aday AW Almarzooq ZI; American Heart Association Council on Epidemiology and Prevention Statistics Committee and Stroke Statistics Subcommittee: 2024 Heart Disease and Stroke Statistics: A Report of US and Global Data From the American Heart Association. Circulation. 149: e347–e913, 2024. 38264914 10.1161/CIR.0000000000001209 PMC12146881

[2] Kirkpatrick JN Mahr C Beckman J Bjelkengren J Dudzinski DM: Responding to ventricular assist device recalls: An ethical guide for mechanical circulatory support programs. ASAIO J. 66: 363–366, 2020. 31045923 10.1097/MAT.0000000000001005

[3] Gilotra NA Stevens GR: Temporary mechanical circulatory support: A review of the options, indications, and outcomes. Clin Med Insigh. 8(suppl 1): CMC.S15718, 2014. 10.4137/CMC.S15718 PMC4317108 25674024

[4] Kirklin JK Pagani FD Kormos RL: Eighth annual INTERMACS report: Special focus on framing the impact of adverse events. J Heart Lung Transplant. 36: 1080–1086, 2017. 28942782 10.1016/j.healun.2017.07.005

[5] Veasey TM Floroff CK Strout SE: Evaluation of anticoagulation and nonsurgical major bleeding in recipients of continuous-flow left ventricular assist devices. Artif Organs. 43: 736–744, 2019. 30868618 10.1111/aor.13456

[6] Figueroa Villalba CA McMullan DM Reed RC Chandler WL: Thrombosis in extracorporeal membrane oxygenation (ECMO) circuits. ASAIO J. 68: 1083–1092, 2022. 34860711 10.1097/MAT.0000000000001605

[7] Baran DA Jaiswal A Hennig F Potapov E: Temporary mechanical circulatory support: Devices, outcomes, and future directions. J Heart Lung Transplant. 41: 678–691, 2022. 35461760 10.1016/j.healun.2022.03.018

[8] Hord EC Bolch CM Tuzun E Cohn WE Leschinsky B Criscione JC: Evaluation of the CorInnova Heart Assist Device in an Acute Heart Failure Model. J Cardiovasc Transl Res. 12: 155–163, 2019. 30604307 10.1007/s12265-018-9854-5 PMC6497617

[9] Letsou GV Bolch CM Hord EC Altman WC Leschinsky B Criscione JC: The CorInnova implantable cardiac assist system for direct cardiac compression. RCM. 23: 211, 2022. 10.31083/j.rcm2306211 PMC11273667 39077181

[10] Letsou GV Bolch CM Hord EC: Mechanical cardiac support with an implantable direct cardiac compression device: Proof of concept. Ann Thorac Surg. 114: 1944–1950, 2022. 35921854 10.1016/j.athoracsur.2022.06.052 PMC9610996

[11] Morley D Litwak K Ferber P: Hemodynamic effects of partial ventricular support in chronic heart failure: Results of simulation validated with in vivo data. J Thorac Cardiovasc Surg. 133: 21–28, 2007. 17198776 10.1016/j.jtcvs.2006.07.037

[12] Punnoose L Burkhoff D Rich S Horn EM: Right ventricular assist device in end-stage pulmonary arterial hypertension: Insights from a computational model of the cardiovascular system. Prog Cardiovasc Dis. 55: 234–243.e2, 2012. 23009919 10.1016/j.pcad.2012.07.008

[13] Doshi D Burkhoff D: Cardiovascular simulation of heart failure pathophysiology and therapeutics. J Card Fail. 22: 303–311, 2016. 26703246 10.1016/j.cardfail.2015.12.012

[14] Griffin JM Borlaug BA Komtebedde J: Impact of interatrial shunts on invasive hemodynamics and exercise tolerance in patients with heart failure. J Am Heart Assoc. 9: e016760, 2020. 32809903 10.1161/JAHA.120.016760 PMC7660772

[15] Burkhoff D Maurer MS Joseph SM: Left atrial decompression pump for severe heart failure with preserved ejection fraction: Theoretical and clinical considerations. JACC Heart Fail. 3: 275–282, 2015. 25770409 10.1016/j.jchf.2014.10.011

[16] Leisman S Burkhoff D: Use of an iPad App to simulate pressure-volume loops and cardiovascular physiology. Adv Physiol Educ. 41: 415–424, 2017. 28679580 10.1152/advan.00204.2016

[17] Burkhoff D Dickstein ML Schleicher T: Harvi-Online. Available at: https://harvi.online. Accessed June 2024.

[18] Kaye D Shah SJ Borlaug BA: Effects of an interatrial shunt on rest and exercise hemodynamics: Results of a computer simulation in heart failure. J Card Fail. 20: 212–221, 2014. 24487087 10.1016/j.cardfail.2014.01.005

[19] Schafer A Burkhoff D Bauersachs J: Haemodynamic simulation and the effect of early left ventricular unloading in pre-shock acute coronary syndrome. ESC Heart Fail. 6: 457–463, 2019. 30861640 10.1002/ehf2.12417 PMC6487719

[20] Kaye DM Byrne M Mariani J Nanayakkara S Burkhoff D: Identification of physiologic treatment targets with favourable haemodynamic consequences in heart failure with preserved ejection fraction. ESC Heart Fail. 7: 3685–3693, 2020. 32902205 10.1002/ehf2.12908 PMC7754909

[21] Artrip JH Yi G-H Levin HR Burkhoff D Wang J: Physiological and hemodynamic evaluation of nonuniform direct cardiac compression. Circulation. 100: II236–II243, 1999. 10567310 10.1161/01.cir.100.suppl_2.ii-236

[22] Artrip JH Wang J Leventhal AR Tsitlik JE Levin HR Burkhoff D: Hemodynamic effects of direct biventricular compression studied in isovolumic and ejecting isolated canine hearts. Circulation. 99: 2177–2184, 1999. 10217660 10.1161/01.cir.99.16.2177

[23] Oz M Artrip J Burkhoff D: Direct cardiac compression devices. J Heart Lung Transplant. 21: 1049–1055, 2002. 12398868 10.1016/s1053-2498(02)00482-5

[24] Sunagawa K Maughan WL Burkhoff D Sagawa K: Left ventricular interaction with arterial load studied in isolated canine ventricle. Am J Physiol. 245: H773–H780, 1983. 6638199 10.1152/ajpheart.1983.245.5.H773

[25] Sunagawa K Sagawa K Maughan WL: Ventricular interaction with the loading system. Ann Biomed Eng. 12: 163–189, 1984. 6507965 10.1007/BF02584229

[26] Alexander J Jr Sunagawa K Chang N Sagawa K: Instantaneous pressure-volume relation of the ejecting canine left atrium. Circ Res. 61: 209–219, 1987. 3621487 10.1161/01.res.61.2.209

[27] Burkhoff D: Explaining load dependence of ventricular contractile properties with a model of excitation-contraction coupling. J Mol Cell Cardiol. 26: 959–978, 1994. 7799451 10.1006/jmcc.1994.1117

[28] Lankhaar JW Rovekamp FA Steendijk P: Modeling the instantaneous pressure-volume relation of the left ventricle: A comparison of six models. Ann Biomed Eng. 37: 1710–1726, 2009. 19554450 10.1007/s10439-009-9742-x PMC3233835

[29] Westerhof N Lankhaar JW Westerhof BE: The arterial Windkessel. Med Biol Eng Comput. 47: 131–141, 2009. 18543011 10.1007/s11517-008-0359-2

[30] Westerhof N Bosman F De Vries CJ Noordergraaf A: Analog studies of the human systemic arterial tree. J Biomech. 2: 121–143, 1969. 16335097 10.1016/0021-9290(69)90024-4

[31] Artrip JH Yi GH Shimizo J: Maximizing hemodynamic effectiveness of biventricular assistance by direct cardiac compression studied in ex vivo and in vivo canine models of acute heart failure. J Thorac Cardiovasc Surg. 120: 379–386, 2000. 10917957 10.1067/mtc.2000.106986

[32] Saxena A Uriel N Burkhoff D: Physiology of blood pump circulation in heart failure. in Karimov JH, (eds), Mechanical Support for Heart Failure: Current Solutions and New Technologies. Cham, Springer International Publishing, 2020, pp. 63–82.

[33] Mann DL Bristow MR: Mechanisms and models in heart failure. Circulation. 111: 2837–2849, 2005. 15927992 10.1161/CIRCULATIONAHA.104.500546

[34] Aranda-Michel E Waldman LK Trumble DR: Computational methods for parametric evaluation of the biventricular mechanics of direct cardiac compression. Artif Organs. 45: E335–E348, 2021. 33908657 10.1111/aor.13974

[35] Hirschvogel M Jagschies L Maier A Wildhirt SM Gee MW: An *in silico* twin for epicardial augmentation of the failing heart. Inter J Num Methods Biomed Eng. 35: e3233, 2019. 10.1002/cnm.3233 31267697

[36] Ingram SN Hager MP Moreno MR Criscione JC: Review of devices and clinical need for non-blood contacting mechanical circulatory support. Appl Eng Sci. 12: 100122, 2022.

