# Supplementary material for: Harvi Cardiovascular Modeling Accurately Predicts Hemodynamic Improvements Produced by a New Direct Cardiac Compression Device
Source: ASAIO J. 2024 Nov 22;71(5):370–8. doi: 10.1097/MAT.0000000000002346 (PMC12039900; doi:10.1097/MAT.0000000000002346)
Supplement: Supplementary file 1 [file mat-71-370-s001.pdf]

Harvi-CorInnova Supplemental Simulation Methods

CorInnova DCC device compression pressure was approximated *in silico* as a trapezoidal waveform with a user-defined inflation rate (“Rise”), a constant peak pressure applied during systole, deflation rate (“Decline”), and a constant negative deflate pressure applied during diastole (“Offset”). The “Amplitude” specified in the simulation was approximated using the average of the *in vivo* assist pressure applied during systole while accounting for the deflate “Offset” during diastole. The “Duration” of assist was simulated using the average pulse pressure duration measured from the real pulses *in vivo*. Finally, the CorInnova version of simulated DCC assist includes the right ventricular scaling factor (RVSF) to simulate the effect of assist on the RV proportional to the effect on the left ventricle as observed *in vivo*; the CorInnova DCC RVSF was constant at 40% for all simulations. The average CorInnova DCC simulation settings are summarized here in Supplemental Materials Table S1. The remaining model inputs for baseline and HF samples are summarized in Supplemental Materials Table S2.

TABLE S1. Summary of CorInnova DCC assist simulation input settings

	**Offset** *ms*	**Delay** *ms*	**Amplitude** *mmHg*	**Rise** *mmHg/ms*	**Decline** *mmHg/ms*	**Duration** *ms*	**RV Scaling Factor**
**Animal Data (mean ± σ) (n=12)**	-3 ± 1	0	22 ± 7	0.2 ± 0.0	0.3 ± 0.1	309 ± 19	40
**PV Loop Simulations**							
**HF+DCC19**	-3	0	22	0.2	0.3	308	40
**HF+DCC30**	-4	0	34	0.3	0.3	308	40

Abbreviations: DCC: direct cardiac compression, RV: right ventricle, PV: pressure-volume, HF: heart failure

TABLE S2. Additional Model Inputs *(mean)*

Parameter group/name	Symbol	Units	Values							
			Baseline	HF						
Heart rate	HR	min^{-1}	109	91						
Stressed blood volume (Preload)	Vol	mL	1430	1740						
**Heart**			**LA**		**LV**		**RA**		**RV**	
			**Baseline**	**HF**	**Baseline**	**HF**	**Baseline**	**HF**	**Baseline**	**HF**
End-systolic elastance	Ees	mmHg/mL	0.72	0.42	2.42	0.90	0.70	0.30	0.65	0.40
Volume axis intercept	Vo	mL	5	5	5	5	5	5	5	5
Exponent for EDPVR	α	mL^{-1}	0.081	0.081	0.054	0.054	0.077	0.077	0.051	0.051
Scaling factor for EDPVR	β	mmHg	0.44	0.44	0.34	0.34	0.44	0.44	0.34	0.34
Time to end-systole	Tmax	ms			226	274			226	274
Time constant of relaxation	t	ms			37	51			37	51
Chamber end-diastolic volume	EDV	mL	50	50			50	50	76	76
Ventricular Wall Thickness	Th	cm			0.9	0.70			0.5	0.40
**Circulation**			**Systemic**		**Pulmonary**					
			**Baseline**	**HF**	**Baseline**	**HF**				
Characteristic impedance	Rc	mmHg*s/mL	0.01	0.08	0.04	0.07				
Vascular resistance	Ra	mmHg*s/mL	0.81	0.66	0.01	0.01				
Vascular compliance	Ca	mL/mmHg	2.60	3.25	20.31	22.45				

Abbreviations: HF: heart failure, LA: left atrium, LV: left ventricle, RA: right atrium, RV: right ventricle, EDPVR: end-diastolic pressure-volume relation
